# Emergency surgery of intra-articular calcaneal fractures using sinus tarsi approach with modified reduction technique

**DOI:** 10.1186/s12891-023-06636-y

**Published:** 2023-06-26

**Authors:** Yanwen Hu, Lucheng Chen, Yaxing Qian, Junjie Wu, Hao Xu

**Affiliations:** 1 Department of Nuclear Medicine, Suzhou Ninth Hospital, 215000 Suzhou, Wujiang, Jiangsu Province People’s Republic of China; 2 Department of Orthopaedics, RuiHua Orthopaedic Hospital of Suzhou, Jiangsu Province, 5, Tayun Road, 215000 WuZhong, People’s Republic of China; 3 Yongding Hospital of Suzhou, 1000, Gaoxin Road, 215000 Wujiang, Jiangsu Province People’s Republic of China; 4 Department of Orthopaedics, Suzhou Yongding Hospital, 1388, Gaoxing Road, 215000 Suzhou, Wujiang, Jiangsu Province People’s Republic of China

**Keywords:** Emergency surgery, Calcaneal fracture, Sinus Tarsi Approach, Visual Analog Scale, Minimally invasive surgery

## Abstract

**Background:**

The purpose of this study was to evaluate emergency surgery of calcaneal fractures using the sinus tarsi approach (STA) with modified reduction technique in terms of complication rates, iconography results and functional outcome.

**Methods:**

We evaluated the outcomes of 26 patients treated in an emergency using STA with modified reduction technique. For that, we assessed Böhler´s angle, Gissane angle, reduction of the calcaneal body, and posterior facet, the visual analog scale (VAS), American Orthopaedic Foot and Ankle Society (AOFAS) score, complications, preoperative time, operative time, and in-hospital time.

**Results:**

Recovery of calcaneal anatomy and articular surface were found at final follow-up. The mean Böhler´s angle at final follow-up were 30.68° ± 3.69°, of which was 15.02° ± 3.88° preoperatively (p < 0.001). The mean Gissane angle at final follow-up were 114.54° ± 11.16° of which was 88.86° ±10.96° preoperatively (p < 0.001). All cases had the varus/valgus angle of the tuber within 5 degrees. At the final follow-up, the mean AOFAS score was 89.23 ± 4.63, and the VAS score was 22.73 ± 6.5.

**Conclusions:**

Emergency surgery using STA with modified reduction technique is reliable, effective, and safe for treatment of calcaneal fractures. This technique can bring good clinical outcomes and a low rate of wound complications, reducing the in-hospital time, costs, and accelerating rehabilitation.

## Introduction

Intra-articular fractures are common but difficult to deal, because of fragile soft tissue and the complicated anatomic structure [1]. Calcaneal fractures are classified as types I to IV according to the number of articular fracture lines using Sanders classification. For over 2500 years, conservative treatment was the only available option due to the high risk of soft tissue without ideal management. At the beginning of the twentieth century, treatment was closed reduction and external immobilization [2]. The traditional management methods of displaced calcaneal fractures are open surgery and internal fixation through extended L-shape lateral approach, which allows a direct reset of the depressed posterior facet fragment and provides excellent iconography results including the Böhler angle (i.e. the angle between the line joining the highest point of the anterior process with the highest point of the posterior facet and the line tangent to the superior surface of the tuberosity) and the Gissane angle (i.e. the angle between the line of the anterior end of the posterior facet to the dorsal edge of the calcaneocuboid facet and the line tangent to the articular surface of the medial posterior facet) [1]. However, there are many wound soft tissue complications about L-shape lateral approach, such as hematoma, infection, wound edge necrosis, and dehiscence [1]. The sinus tarsi approach (STA) is one of the most effective and popular minimally-invasive methods, with superior direct access to the posterior facet and with few soft tissue complication [3]. STA has some difficulty during operation, such as limited field of vision and inadequate space to reduce calcaneal medial column displacements. Occasionally surgeons add a medial incision or extended incision to reduce the displaced fragments. Using clamps and Steinmann pins to reduce fragments are often insufficient, especially in complex fractures. Therefore, we used a modified reduction technique to assist STA, which can reduce medial fragments percutaneously from the lateral side without elongating incision.

Intra-articular calcaneus fractures can be treated 1–2 weeks before surgery in the extended L-shape lateral approach, and 3–5 days in STA because of swelling or blister formation [1, 3]. It often elongates the in-hospital time, raises hospitalization costs and it can not bring quick rehabilitation to patients. Few studies reported about emergency surgery of calcaneal fractures using STA.

The aim of our study was to assess emergency surgery of calcaneum fractures using STA with modified reduction technique in terms of complication rates, iconography results and functional outcome. To the best of our knowledge, it is the first time to report an emergency surgery using STA with modified reduction technique for calcaneal fractures. We hypothesized that emergency surgery using STA with modified reduction technique can provide a low rate of complications and good clinical outcomes.

## Materials and methods

### Subjects

This study retrospectively reviewed 28 consecutive patients (28 feet) with displaced intra-articular calcaneal fractures. Patients underwent emergency surgery using STA with modified reduction technique at 2 different institutions, between January 2019 and January 2022. Institutional review board approved this study. All patients gave oral and written consent in this study. Inclusion criteria were: (i) fresh and closed fractures injured within 24 h; (ii) Sanders type II or III intra-articular fractures; and (iii) fractures that underwent STA with modified reduction technique. Patients with diabetes, open fractures or other peripheral vascular diseases, extra-articular fractures, and long-term smoking status were excluded. Two patients were lost during follow-up time. A total of 26 feet of 26 patients were included in this study. The mean age of patients was 43.04 ± 8.67 (28–56) years; 25 were male and 1 were female. The mean body mass index was 24.99 ± 2.23 (range 20.46 to 29.41) kg/m^2^. All patients were injured by falls. The right side was involved in 11 patients and the left side in 15 patients. There were 14 type II and 12 type III calcaneal fractures. All patients were operated by the same surgeons who had experience of performing foot surgery for at least ten years.

### Standard operative technique

**Fig. 1:**
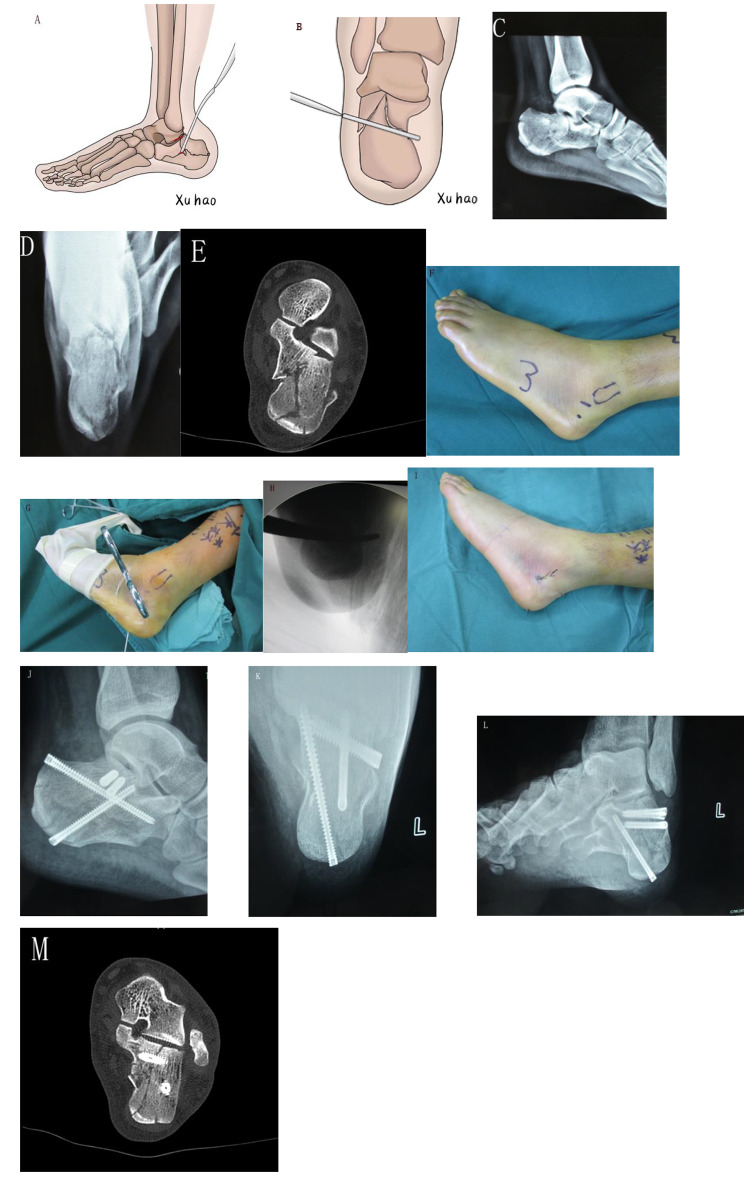
A 40-year male treated with STA. **A**, **B** Diagrammatic depiction of STA with modified reduction technique. **C**, **D** X-ray showed a calcaneous fracture. Lateral view and axial view. **E** Preoperative images showing a Sanders type II intra-articular calcaneal fracture. **F** Operative scheme showing the STA incision. **G**, **H** Operative pictures and radiographs showing STA combined with modified reduction technique. **I** Postoperative picture of the incision. **J**, **K**, **L**, **M** Postoperative images after surgery

Patient was placed laterally, then a tight tourniquet was placed at the thigh after spinal or general anesthesia. A 1–2 cm incision was performed from the tip of the fibula to the base of the fourth metatarsal. Careful dissection was performed when the peroneal tendons and the sural nerve were met. Another lateral incision of 0.5 cm was performed under the STA incision. A vessel forceps or a periosteal elevator was inserted in the incision from the lateral fragment to the bottom of the medial wall of the calcaneus. Then, the medial sustentacular fragment was elevated over the calcaneal tuberosity. The outward displaced calcaneal tuberosity, varus deformity, calcaneal height, and medial wall overlap were corrected, then 1 or 2 2.0-mm K-wires temporarily fixed the fragments. One or two 4.5-mm screws were placed from lateral posterior facet to the medial sustentacular fragment. From the calcaneus tubercle towards the anterior process, one or two 5.5-mm cannulated screws were inserted through the calcaneus length. One screw was placed from the calcaneus tubercle to the sustentaculum tali or another screw was placed from the calcaneus tubercle to the lateral joint fragments. One rubber drain was inserted in the wound before closing the incision (Fig. 1).

### Postoperative management

The operated foot of each patient was elevated to alleviate swelling. All patients were allowed to have a full range of ankle motion the next day. After six weeks, patients could start partial weight-bearing. Full weight-bearing was encouraged after bony union being observed on the radiograph around 12 weeks postoperatively. We followed-up the fractures for 3, 6, 12 months after surgery and then yearly thereafter until the final follow-up. The anatomical parameters including the Böhler´s angle and the Gissane angle were measured. Postoperative CT was performed after surgery and 1 year after surgery. Post-operative CT scans were performed to observe flatness of the calcaneal posterior facet. A step < 1.0 mm of posterior facet was considered an “anatomic reduction”. A step between 1 and 3 mm was deemed “near anatomic reset”. A step between 3 and 5 mm was deemed “approximate reset”. A step > 5 mm was considered “failed reset” [4]. The axial X ray of calcaneum was performed to survey the varus/valgus angle of the calcaneum. An angulation < 5 degrees was deemed “normal”. The functional outcomes were assessed by the American Orthopedic Foot and Ankle Society (AOFAS) score and the 10 cm visual analog scale (VAS) at the final follow-up.The AOFAS score was graded as excellent (90), good (80), fair (70), and poor (< 70). Time to operation, operative time, in-hospital time, and wound complications were recorded at post-operation or at the last follow-up. All measurements were taken in the same period by two independent surgeons, who did not participate in the surgeries or patient care. The other assessors conducted the statistical analyses and follow-up evaluations, and did not participate in the surgeries or patient care.

### Statistical analysis

Descriptive statistics were calculated including means and standard deviations for continuous variables. All continuous data were tested for normal distribution using the Shapiro-Wilk test. Paired t test was used for radiographic outcomes pre-operatively and post-operatively. Statistical significance was defined as p < 0.05. Statistical analyses were conducted by SPSS 20.0 software (SPSS Inc., Chicago, IL, U.S.A.).

## Results

The median time from injury to surgery was 6 (range 4 to 12.25) hours. The mean hospital stay was 9.8 ± 2.83 (range 5 to 18) days, and the mean operative time was 67.12 ± 18.39 (range 40 to 110) minutes. The median follow-up period was 25.5(range 22.75 to 32.5) months. Demographic data are shown in Table 1.

**Fig. 2:**
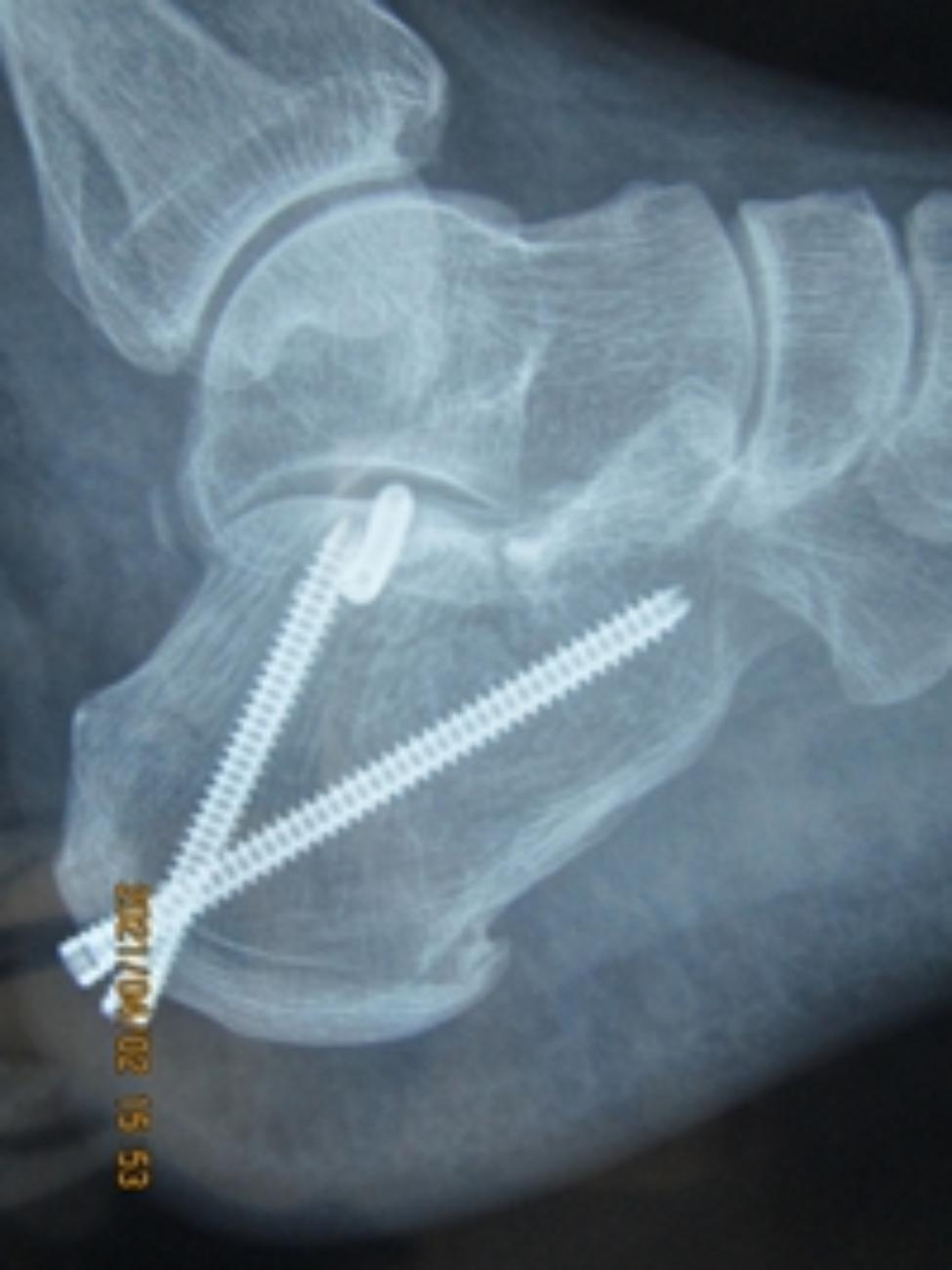
X-ray of incorrect screw placement affected subtalar joint

There was a superficial wound infection case, which was cured by regular wound care and given oral antibiotics for 2 weeks. There was one case of wound edge necrosis that cured with regular wound care within 2 weeks. One patient had transitory sural nerve paralysis, which recovered spontaneously after 2 weeks. One patient had incorrect screw placement, which affected subtalar joint, but the patient did not have uncomfortable feeling (Fig. 2). There were two case of prominent screw heads with no tenderness, and there was no cases with significant subtalar arthritis or reduction loss at the final follow-up.

There were obvious changes in radiographic parameters at postoperatively and at the final follow-up compared to preoperative condition (Table 2). The mean Böhler´s angle postoperatively and at final follow-up were 30.98° ± 3.77° (range 22.1° to 36.8°) and 30.68° ± 3.69° (range 23.4° to 37.6°), which were much higher than 15.02° ± 3.88° (range 9.4° to 25.7°) preoperatively (p < 0.01). The mean Gissane angle postoperatively and at final follow-up were 115.24° ± 12.02° (range 97.2° to 135.9°) and 114.54° ± 11.16° (range 95.6° to 131.1°), which were much higher than 88.86° ±10.96° (range 69.7° to 110.5°) preoperatively (p < 0.01). We assessed restoration of the posterior facet at the final follow-up. A near anatomic reduction of the articular surface (1–3 mm step) was found in 2 cases. No cases of an approximate or failed anatomic reset of articular surface were found. All cases had the varus/valgus angle of the tuber within 5 degrees.

**Table 1:** Patients’ demographics

Parameter	Value
Sex	
Male Female	25 1
Mean age(y)	43.04 ± 8.67
Fracture side	
Right	11
Left	15
Injury mechanism	
Fall from height	26
Sanders classification	
Type II	14
Type III	12
Mean BMI (kg/m2)	24.99 ± 2.23
Median follow-up ( mo )	25.5
Mean operative time (min)	67.12 ± 18.39
Median time to surgery (h)	6
mean hospital stay (d)	9.8 ± 2.83
Complications	
Superficial infection	1
Sural nerve paralysis	1
Wound edge necrosis	1
Incorrect screw placement	1
Prominent screw heads	2

**Table 2:** The results of the radiographic measurements (N = 26 feet in 26 patients)

Outcome	Preoperative	Postoperative	p Value
Böhler angle	15.02° ± 3.88°	30.98° ± 3.77°	<0.01
Gissane angle	88.86° ±10.96°	115.24° ± 12.02°	<0.01

Data presented as mean ± standard deviation

All cases had bony union, and after about 12 weeks the fractures were healed. Most patients returned to daily living with normal activities (92.3%). At the final follow-up, the mean AOFAS score was 89.23 ± 4.63 (range 80 to 96); 21 cases were excellent, 4 cases were good, and 1 case was fair, with an excellent/good rate of 96.2%. At the final follow-up, the VAS score was 22.73 ± 6.5 (range 11 to 33).

## Discussion

Extended L-shape lateral approach can restore the normal calcaneal anatomy. However, it is accompanied with high risk of wound complications [5, 6]. Minimally invasive techniques have been popular, which can bring more rapid wound healing, fewer soft tissue complication, shorter in hospital days and less postoperative pain [7]. Many reports have proved that minimally invasive fixation can provide the efficacy function and fewer complications [8, 9]. However, minimally invasive techniques using STA, usually performed after several days before surgery, elongates the hospital time and delays rehabilitation [3]. There are still few reports about emergency surgery using STA.

The outcomes of our study validated the hypothesis that emergency surgery using STA with modified reduction technique can provide good clinical outcomes. Radiographic outcomes revealed that Böhler’s angle and Gissane angles were significantly changed at the last follow-up. Only one patient had wound edge necrosis. Biz C, et al. [10] conducted a retrospective study with 104 calcaneal fractures cases, they found that there were no statistical significance in clinical outcomes between traditional open technique group and percutaneous surgical procedures group. Baca E, et al. [11] reviewed 114 cases of surgically treated calcaneal fractures with a fast, less complicated, and modified percutaneous technique; they found modified percutaneous fixation can give good results. These outomes are similar with the findings of our study.

Some studies have reported soft tissue complication using standard STA with plating [12, 13]. Dissection and plate insertion can damage soft tissue, and it might induce relatively high complication rate. Hence, STA for calcaneal fractures is usually performed after several days. The waiting time before surgery raises the cost and rehabilitation time. In our study, there was only one case of incision complication, and all patients had anatomic reduction. We think there are some critical factors about the surgery. Inserting a standard calcaneal plate using STA needs a bigger place to be manipulated. Surgeons often extend the wound proximally or posteriorly, which can damage the lateral calcaneal branch of the peroneal artery and could increase the rate of skin necrosis Using traditionary percutaneous instruments, including Steinmann pins and reduction clamps, but it can induce inadequate reduction. Some surgeons conducted a medial incision to manipulate the medial fragment, which can damage the medial nerve and vessels [14]. In our previous study, STA with modified reduction technique can effectively reduce the medial wall displacement, without extending the incision or adding another medial incision [15]. In our study, only a 1–2 cm incision was used in percutaneous screw fixation, and it is less invasive and may acquire fewer wound complications. During foot swelling, a small incision with emergency can decompress the swelled foot. Emergency surgery can decrease the hospital time and accelerate rehabilitation. In our study, all patients were found to have bony union. The VAS score was 0.82 ± 0.95 and the AOFAS score was 80.08 ± 5.44 at the last follow-up.

Many studies have reported clinical and biomechanical outcomes that screw fixation has similar efficacy to plate fixation. Pitts, CC et al. [16] reviewed that 51 patients underwent ORIF with cannulated screws alone, and 23 patients underwent ORIF with a plate. They found there was no difference in restoration of anatomical parameters, either method effectively increased radiographic parameters. Guo, C et al. [17] performed a study to compare plate fixation with screw fixation for calcaneum fractures using sinus tarsi approach. They found that both fixation methods had similar clinical outcomes. Nelson et al. [18] conducted a cadaver study where they found that screw fixation and plate fixation had similar biomechanical stability and proved that screw fixation method is effective for a minimally-invasive calcaneum fracture surgery. Ni et al. [19] compared cannulated screws, K-wires, absorbable screws, and plate fixation. They found that plate-screw and cannulated screws had similar stiffness for calcaneal fractures. In our study, the Gissane and Böhler´s angle were well restored by screw fixation, and no cases of reduction loss during follow-up period.

There were some limitations in our study. First, the number of patients was limited, particularly cases of Sanders type IV fractures were excluded, and the follow-up duration was limited. Second, a control group and prospective study should be designed in future.

## Conclusions

Emergency surgery using STA with modified reduction technique is effective and safe for calcaneum fractures. We believe that emergency surgery using STA with modified reduction technique could be an alternative method for calcaneal fracture. Using this method, it can give ideal clinical outcomes, and a low rate of wound complications, reducing the in-hospital time, costs and accelerating rehabilitation.

## Data Availability

The datasets used and/or analyzed during the current study are available from the corresponding author on reasonable request. Readers can access the data and material supporting the conclusions of the study by contacting Hao Xu at 38077474@qq.com.
